# A DNA methylation-based algorithm for diagnosing rheumatoid arthritis

**DOI:** 10.1186/s13075-025-03649-x

**Published:** 2025-10-17

**Authors:** Espen Riskedal, Astanand Jugessur, Silje Watterdal Syversen, Cathrine Lund Hadley, Jennifer R. Harris, Maria Dahl Mjaavatten, Joe Sexton, Janis Neumann, Gina Hetland Brinkmann, Guro Løvik Goll, Grethe-Elisabeth Stenvik, Håkon Bøås, Arne Søraas, Karl Trygve Kalleberg, Siri Lillegraven, Espen A. Haavardsholm

**Affiliations:** 1 Age Labs AS, Gaustadalléen 23A, Oslo, Norway; 2 Centre for Fertility and Health, Norwegian Institute of Public Health, Oslo, Norway; 3 Department of Global Public Health and Primary Care, University of Bergen, Bergen, Norway; 4 Center for treatment of Rheumatic and Musculoskeletal Diseases (REMEDY), Diakonhjemmet Hospital, Box 23 Vinderen, Oslo, 0370 Norway; 5 Faculty of Medicine, University of Oslo, Oslo, Norway

**Keywords:** Rheumatoid arthritis, Classification algorithm, Seronegative, Epigenetics, DNA methylation

## Abstract

**Background:**

Rheumatoid arthritis (RA), particularly seronegative disease, is difficult to diagnose early, which can delay treatment initiation. This study aims to develop a binary DNA methylation (DNAm)-based algorithm to diagnose RA.

**Methods:**

Three datasets (discovery, training, holdout) were constructed from DNAm profiles from 1366 persons (treatment-naïve RA, other inflammatory/autoimmune diseases, healthy individuals). DNAm features that differentiate RA from other inflammatory/autoimmune diseases and healthy individuals were identified using the discovery set. Our classification algorithm was developed using machine learning techniques in the training set. Its diagnostic performance, with and without serological status, was evaluated in the holdout set containing RA cases (15 seropositive, 6 seronegative) and controls (14 other arthritides, 11 healthy individuals).

**Results:**

Our algorithm included 391 DNAm features. Combined with serological status, it classified RA from controls in the holdout set with the following performance: sensitivity 0.90 [95% CI: 0.70–0.99], specificity 0.88 [95% CI: 0.69–0.97], and AUC 0.96 [95% CI: 0.91–1.00]. Its performance in classifying patients with seronegative RA versus those with other arthritides was: sensitivity 0.83 [95% CI: 0.36–1.00], specificity 0.79 [95% CI: 0.49–0.95], and AUC 0.81 [95% CI: 0.61–1.00].

**Conclusions:**

The present DNAm-based classification algorithm may be clinically useful for the early diagnosis of RA, especially in seronegative patients, which currently often poses a diagnostic challenge.

**Supplementary Information:**

The online version contains supplementary material available at 10.1186/s13075-025-03649-x.

## Background

Early identification of rheumatoid arthritis (RA) can be challenging, particularly for seronegative disease. The pathophysiology of RA is not completely understood [1, 2], and no single test or gold standard exists to confirm the diagnosis. Arthritis can be caused by various factors, ranging from infection related to crystal deposition, or it may be an early sign of chronic inflammatory joint diseases such as RA. A clinical diagnosis of RA is made based on a set of typical findings and symptoms, including the distribution of arthritis, the patient’s history, inflammatory markers and serology assessed by anti-citrullinated peptide antibodies (ACPA) and rheumatoid factor (RF) [1, 3, 4]. However, diagnostic differentiation is often difficult in the early stages of inflammatory arthritis [3, 5].

Early arthritis clinics were established in the early 2000s, contributing to the understanding of early RA and providing the basis for the development of improved RA classification criteria [4, 6]. After the introduction of the American College of Rheumatology (ACR)/ European Alliance of Associations for Rheumatology (EULAR) 2010 classification criteria, patients with negative serology must have at least ten joints involved to fulfill the classification criteria for RA [4, 7]. This might lead to a larger fraction of seronegative patients being initially classified as undifferentiated arthritis.

Overall, the ACR/EULAR 2010 criteria led to an improved diagnostic process for patients with positive serology, but the benefit for patients without positive serology is less clear [8]. Patients classified as seronegative RA exhibit a higher burden of inflammation compared to patients with seropositive RA [9, 10]. A study reported that the median time from first joint swelling to the clinical diagnosis was 187 days in seronegative patients compared to only 11 days in seropositive patients. Furthermore, up to 75% of seronegative patients were not classified early enough based on the 2010 criteria [8, 11]. As novel data indicate that early treatment, even prior to clinical arthritis is potentially valuable [12, 13], this delayed diagnosis and treatment of seronegative RA patients can result in patients missing the window of opportunity for treatment, resulting in a poorer prognosis [11, 14], less chance of drug-free remission [15], and higher treatment costs [16]. Early identification of seronegative RA patients therefore remains an unmet clinical need.

Peripheral immune cells are known to be involved in the pathology of RA [17, 18], and DNA methylation (DNAm) changes in these immune cells have been reported to be associated with RA [19–21]. Although genetics plays a role in disease development, monozygotic twins exhibit a concordance rate of only 12–15% for RA [2, 22, 23], and even lower for seronegative RA. These findings imply that non-genetic factors may explain a larger amount of variance in disease susceptibility. Our hypothesis was that common DNAm changes present in both seropositive and seronegative patients may present an opportunity to develop a diagnostic test for better detection of both seropositive and seronegative RA. Accordingly, the aim of the current study was to develop a DNAm-based algorithm for the early diagnosis of RA based on methylation data derived from whole blood samples and to validate the diagnostic performance of the algorithm in combination with existing serological markers.

## Materials and methods

### Study population

**Fig. 1:**
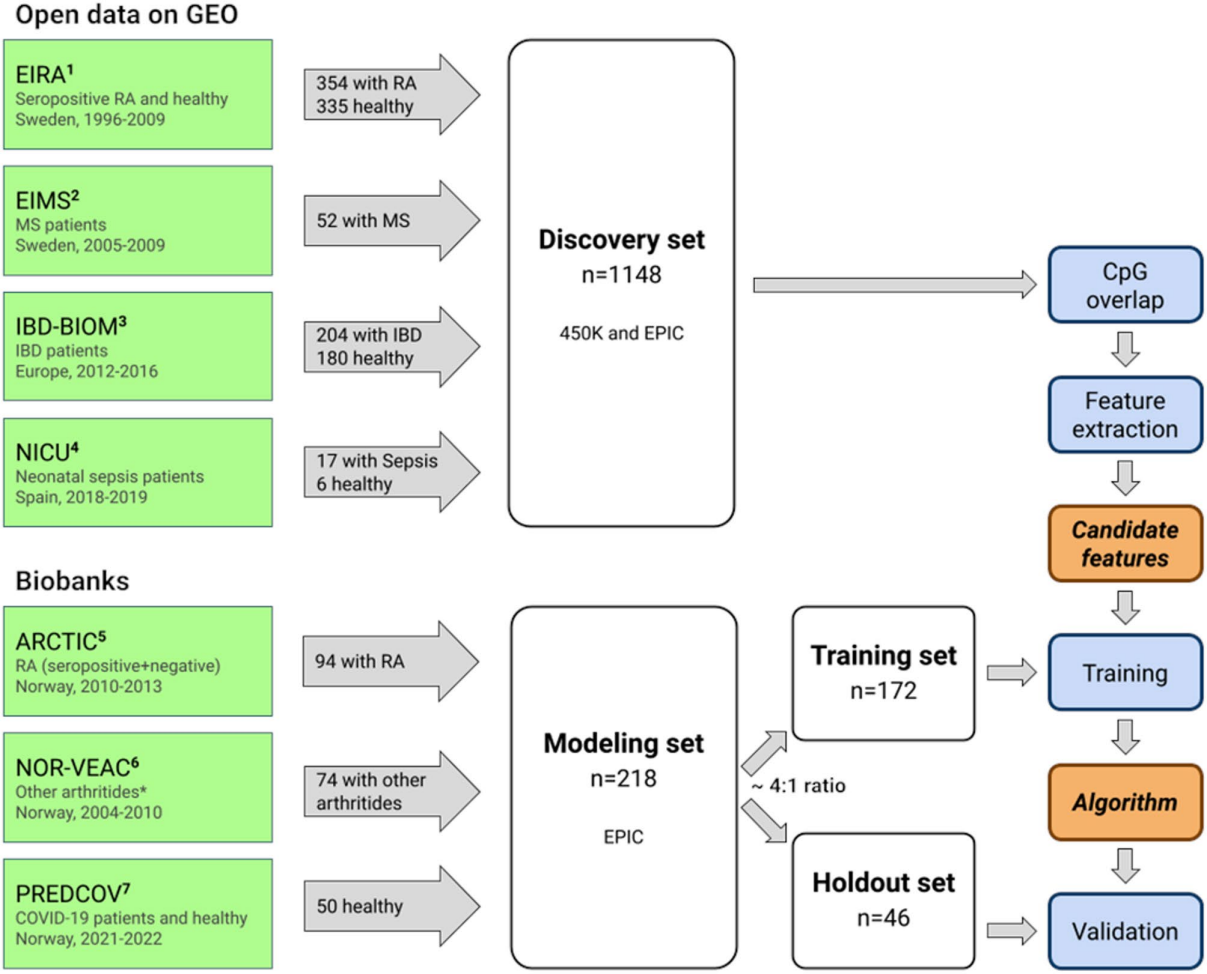
Analytical pipeline applied to the discovery and modeling sets. This study is based on two main datasets: a discovery set consisting of 1148 individuals and a modeling set consisting of 218 individuals. Biobanks are highlighted in green, datasets in white, methods in blue, and output in orange. The publicly-available data from the GEO repository were combined into a discovery set which was subsequently used to identify DNAm features significantly differentially methylated between cases and controls. The modeling set was randomly split into a training set (n = 172) and a holdout set (n = 46) in an approximately 4:1 ratio, and used to train and validate our classification algorithm, respectively. 450K and EPIC refer to the Illumina arrays used to generate the DNAm data. Acronyms: ^1^EIRA = Epidemiological Investigation of RA; ^2^EIMS = Epidemiological Investigation of Multiple Sclerosis; ^3^IBD-BIOM = Inflammatory Bowel Disease Biomarkers Program; ^4^NICU = Neonatal Intensive Care Unit Study; ^5^ARCTIC = Aiming for Remission in Rheumatoid Arthritis; ^6^NOR-VEAC = Norwegian Very Early Arthritis Clinic; ^7^PREDCOV = Predict Covid

We assembled two datasets: a *discovery* set and a *modeling* set (Fig. 1). The discovery set was used to identify DNAm features that can discriminate between cases (RA) and controls (non-RA). The modeling set was used to develop the classification algorithm and was further split into a *training set* and a *holdout set* (Fig. 1).

#### Discovery set

The discovery set (*n* = 1148) was used for feature extraction, meaning the selection of DNAm features, specifically cytosine-phosphate-guanine (CpG) sites, associated with RA. The discovery set comprised 354 treatment-naïve seropositive RA patients, 521 healthy individuals, and 273 individuals with inflammatory bowel disease, multiple sclerosis or sepsis (Table 1; Fig. 1). These data were obtained from the following four publicly available DNAm datasets from the Gene Expression Omnibus (GEO) database (https://www.ncbi.nlm.nih.gov/geo/*)*: The Swedish “Epidemiological Investigation of RA” (EIRA) study [24], the Swedish “Epidemiological Investigation of Multiple Sclerosis” (EIMS) study [25], the European “Inflammatory Bowel Disease Biomarkers Programme” (IBD-BIOM) study [26], and the Spanish “Neonatal Intensive Care Unit Study” (NICU) study [27]. For EIRA, EIMS and IBD-BIOM, DNAm data were generated using the Illumina HumanMethylation450K BeadChip (Illumina, San Diego, USA), but the array type used for NICU was the first version of the Illumina MethylationEPIC BeadChip. From each cohort, we included all patients for whom DNAm data from whole blood were available (Fig. 1).

**Table 1:** Characteristics of the study populations

Datasets	Healthy controls (*n* = 562)	Seropositive RA (*n* = 416)	Seronegative RA (*n* = 32)	Other arthritides (*n* = 74)	IBD (*n* = 204)	MS (*n* = 52)	Sepsis (*n* = 17)	Total (*n* = 1366)
**DISCOVERY**	*(n = 521)*	*(n = 354)*			*(n = 204)*	*(n = 52)*	*(n = 17)*	*(n = 1148)*
Age, median (Q1, Q3)	53 (37, 62)	57 (47, 62)			37 (28, 52)	40 (34, 47)	0 (0, 0)^**1**^	
Female, n (%)	332 (64%)	253 (71%)			94 (46%)	52 (100%)	4 (24%)	
**MODELING**								
**Training**	*(n = 39)*	*(n = 47)*	*(n = 26)*	*(n = 60)*				*(n = 172)*
Age, median (Q1, Q3)	55 (41, 62)	47 (40, 58)	55 (46, 66)	43 (34,57)				
Female, n (%)	25 (64%)	34 (72%)	13 (50%)	33 (55%)				
**Holdout**	*(n = 11)*	*(n = 15)*	*(n = 6)*	*(n = 14)*				*(n = 46)*
Age, median (Q1, Q3)	53 (38, 66)	54 (44, 60)	66 (59, 74)	36 (26, 54)				
Female, n (%)	4 (36%)	11 (73%)	5 (83%)	5 (36%)				
ACPA positive, n (%)		13 (87%)	0 (0%)	0 (0%)				
RF factor positive, n (%)		14 (93%)	0 (0%)	0 (0%)				
Symptom duration months, median (Q1, Q3)		4.7 (1.8, 12.2)	2.5 (2.4, 6.7)	2.1 (1.8, 3.4)				
DAS-28 at baseline median (Q1, Q3)		4.3 (3.9, 4.8)	5.1 (3.9, 5.8)	4.6 (3.9, 5.6)				

The median and IQR values are provided for age, symptom duration, and DAS-28 scores. For the remaining variables, the total number and percentages are provided. The “Other arthritides” category included psoriatic arthritis, reactive arthritis, and undifferentiated arthritis

^**1**^Data from newborns

Abbreviations: IBD = Inflammatory Bowel Disease; MS = Multiple Sclerosis; DAS-28 = Disease Activity Score using 28 joint counts

#### Modeling set

We created a modeling set (*n* = 218) for training and validation of the classification algorithm (Table 1; Fig. 1). DNAm data was generated from whole blood samples from cases (seropositive and seronegative RA) and controls (other arthritides and healthy individuals). Samples from patients with RA and other arthritic diagnoses came from the Norwegian “Aiming for Remission in RA” (ARCTIC) study [28] and the “Norwegian Very Early Arthritis Clinic” (NOR-VEAC) study [29]. Healthy controls were identified from the Norwegian “Predict COVID” (PREDCOV) study [30]. All patients and controls were treatment-naïve for disease-modifying antirheumatic drugs (DMARDs) at the time of sampling.

In NOR-VEAC, the diagnosis of RA was defined as the clinical diagnosis made by the treating rheumatologist. In ARCTIC, all included patients fulfilled the ACR/EULAR 2010 criteria for RA with indication for DMARD treatment [28]. In NOR-VEAC, patients with unresolved arthritis after two years were labeled as undifferentiated arthritis if they had not received a more specific diagnosis. All healthy controls answered a self-assessment questionnaire when they were included in the PREDCOV study, where they were asked about disease status and whether they were on immunosuppressant drugs.

**Fig. 2:**
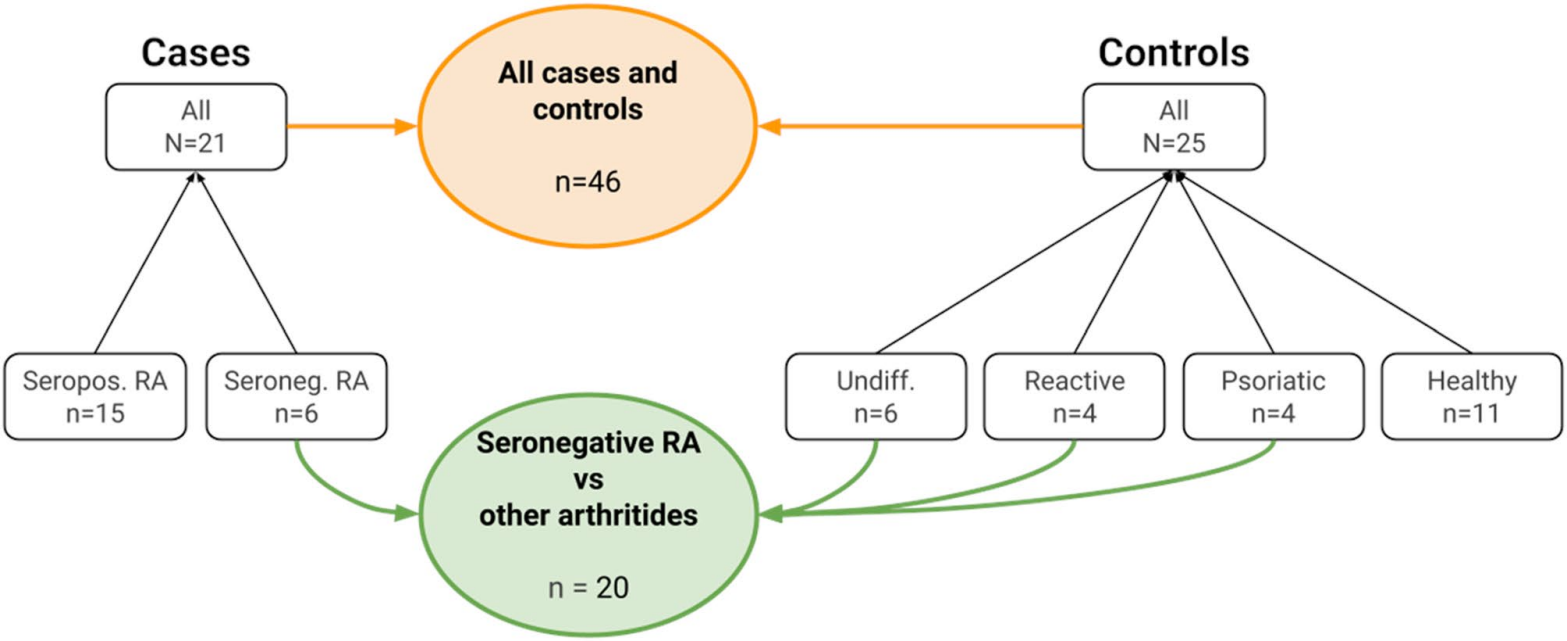
Cases and controls in the holdout set and the subset of seronegative RA compared to other arthritides. This figure shows the breakdown of the samples used in the holdout set and the subset of seronegative RA vs. other arthritides. Cases are shown on the left, controls on the right, and both the holdout set and the subset in the middle. As the label implies, the “*All cases and controls*” holdout set includes all the cases and controls (n = 46). The “*Seronegative RA vs other arthritides*” subset includes seronegative RA, undifferentiated arthritis, reactive arthritis, and psoriatic arthritis (n = 20)

The modeling set was further divided into a training (~ 80%, *n* = 172) and holdout set (~ 20%, *n* = 46) (Table 1; Fig. 1, and Fig. 2). The 80:20 ratio is commonly used among data scientists for this type of analysis [31]. Figure 2 outlines how the holdout set consists of a case (*n* = 21) and control group (*n* = 25), with the case group containing seropositive (*n* = 15) and seronegative (*n* = 6) cases, and the control group containing individuals with undifferentiated arthritis (*n* = 6), reactive arthritis (*n* = 4) and psoriatic arthritis (*n* = 4), as well as healthy individuals (*n* = 11). Note that both the training and holdout set were stratified by age, sex, and diagnosis to ensure a balanced distribution. The training set was used to train the algorithm, while the holdout set was used to validate its diagnostic performance.

### Assessments

#### Serological markers

In the ARCTIC and NOR-VEAC studies, ACPA and RF status were recorded at the first visit to the hospital. RA patients negative for both RF and ACPA were categorized as seronegative RA. The healthy controls from the PREDCOV study did not have ACPA and RF status measured, but were for analytical purposes set to negative serology status.

#### DNAm data

Public DNAm data in the GEO repository were used to create the discovery set (Fig. 1). For the modeling set, DNA was extracted from 200 µl of EDTA-blood from biobanked samples from ARCTIC, NOR-VEAC, and PREDCOV, using the QIAsymphony DSP DNA Mini Kit (QIAGEN, Venlo, Netherlands). The Zymo EZ-96 DNA Methylation-Lightning MagPrep kit (Zymo Research, Irvine, CA, USA) was used for bisulfite conversion of the extracted DNA. DNAm profiles were then generated using the Illumina MethylationEPIC BeadChip (Illumina, San Diego, USA). To minimize batch effects, all samples in the modeling set were randomized and processed in a single batch at Life & Brain GmbH, Bonn, Germany. For extensive details on how the DNAm data were processed, including the extraction of raw signal intensity data from the IDAT files, background correction and normalization, and data harmonization across the different DNAm arrays, see Supplementary Text 1 (under “Bioinformatics methods”).

### Analysis of DNAm data and algorithm creation

**Fig. 3:**
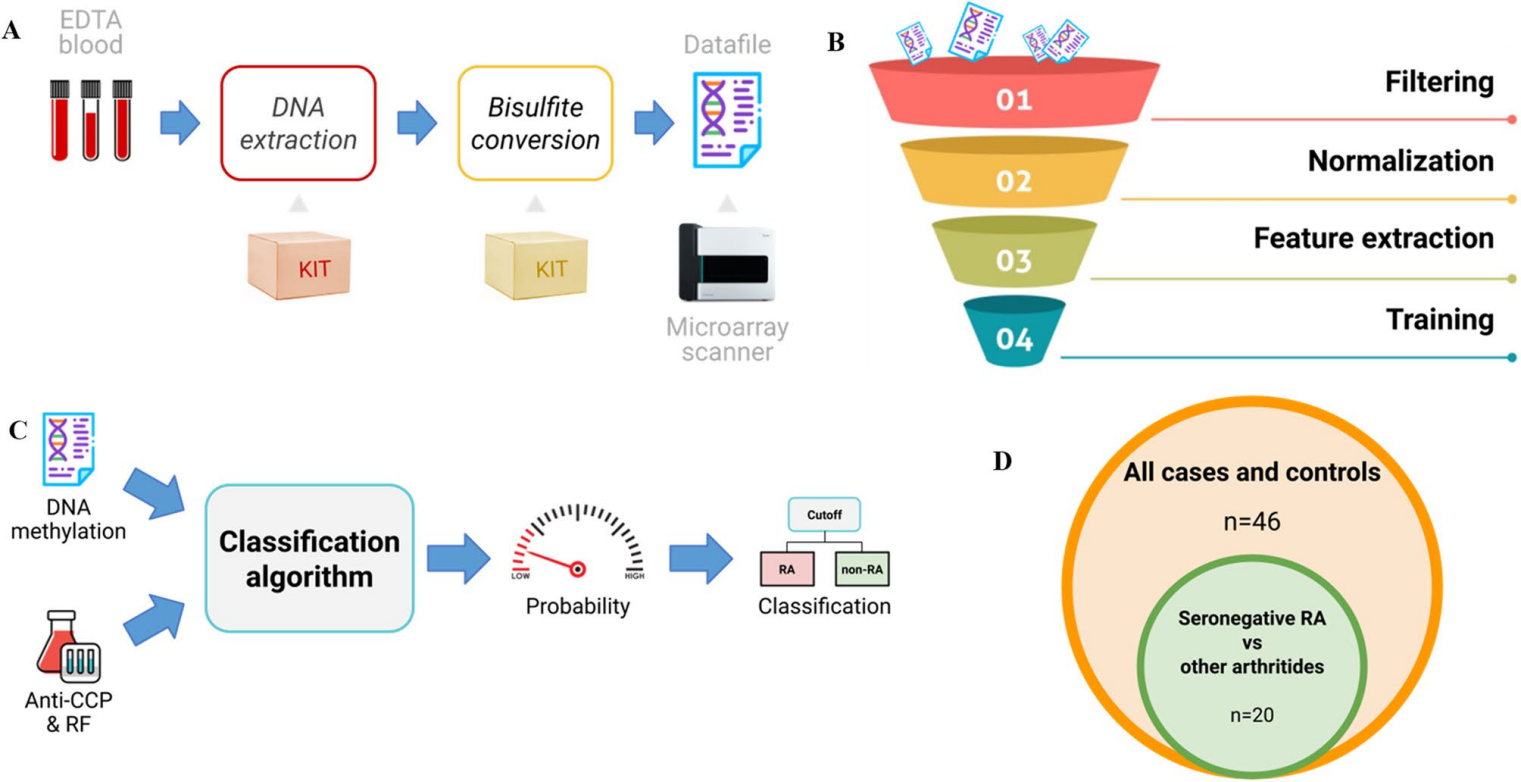
Overview of the analytical strategy used in this study. **A**: DNAm data were derived from EDTA-blood and further processed for DNAm measurement on a microarray scanner. **B**: Individual steps in the machine learning funnel where DNAm data undergo data filtering and normalization. Feature extraction in the discovery set and training in the training set. **C**: A classification algorithm, where methylation levels of specific DNAm features are combined with serology measures (ACPA and RF) to generate a probability score for RA and a classification based on a selected cutoff. **D**: Holdout set used to validate the classification algorithm

Figure 3 provides an overview of the analyses performed in this study. For a more detailed account of how the individual steps in the analyses were carried out, see Supplementary Text 1. The analytic pipeline included the following steps: (A) DNAm data were derived from blood; (B) background correction and normalization were applied to all the DNAm data. DNAm features suitable for discriminating between RA and non-RA were identified using a combination of epigenome-wide association studies (EWAS) and stability selection in the discovery set; (C) candidate algorithms were developed from cross-validation training using elastic net logistic regression on the training set, and the probability scores from the algorithms were dichotomized by applying a cutoff equal to Youden’s J statistic [32]; (D) the out-of-sample performance of the best performing classification algorithm was validated in the holdout set.

We also conducted gene-enrichment and pathway analyses to explore the biological significance of the DNAm features included in our algorithm. For methodological details, see the section on “Pathway and gene-enrichment analysis” in Supplementary Text 1. The purpose of the gene-enrichment analysis was to identify genes with which the DNAm features were associated, while the pathway analysis was to explore the relationship between these genes in biological processes. The results of these analyses are presented in Supplementary Tables 1 and 2.

### Statistical analysis of diagnostic performance

The Student’s t-test was used to assess whether there was a significant difference in the mean algorithm probability score between cases and controls. The diagnostic performance of the algorithm as a continuous score was evaluated using the area under the receiver-operating curve (AUC). Performance metrics, such as sensitivity, specificity, balanced accuracy, and likelihood ratios, were used to gauge the performance of our algorithm. The Shapiro-Wilk normality test was used to assess normality. For normally-distributed variables, Pearson’s product-moment correlation was used to assess the relationship between the algorithm and other serological markers, and for non-normally distributed variables, Spearman’s rank correlation was used instead. *P*-values below 0.05 were considered statistically significant.

Supplementary Text 2 provides details on additional analyses performed to assess algorithm performance. This included comparing our algorithm to three alternative classification algorithms in which age, sex, serology, and DNAm features were included or excluded.

### Patient and public involvement

Although we discussed the study with patients, we did not directly involve them or the public in the design, conduct, reporting, or dissemination plans. The bulk of the work took place during the COVID-19 pandemic, which limited both patient and public involvement in the study.

## Results

### Patient characteristics

Characteristics of the study participants varied across groups, reflecting the different selection criteria (Table 1). Participants with other arthritides were younger than the controls and RA patients.

### Feature extraction

In the discovery set, we identified DNAm features suitable for discriminating between RA and non-RA using a combination of EWAS and stability selection. The EWAS identified 58,236 DNAm features after Holm-Bonferroni correction, while stability selection identified 474 DNAm features with a selection probability higher than 0.1. In total, these methods yielded 58,538 unique DNAm features, which were used when training candidate algorithms on the training set.

### The classification algorithm

The training set was used to train several candidate algorithms. The algorithm that yielded the highest AUC score during training was selected for validation in the holdout set. This classification algorithm consists of 391 DNAm features in combination with ACPA and RF status.

### DNAm features of the algorithm

Of the 391 DNAm features included in the algorithm we investigated the genes associated with the 20 most important DNAm features (Supplementary Table 3). Even though none of them were significantly associated with genes known to be linked to RA, a disproportionately large number of them were genes coding for zinc finger transcription factors that are essential for transcriptional regulation [33]. We also used the GO and KEGG databases to investigate the top 10 pathways associated with the 391 DNAm features (CpGs) included in the algorithm (Supplementary Tables 1 and 2). Several of the pathways reported by KEGG were related to immune response and autoimmune disorders; notably, (i) *path: hsa05168*, which is related to herpes simplex virus 1 infection, (ii) *path: hsa05322* to systemic lupus erythematosus (SLE), (iii) *path: hsa05321* to IBD, and (iv) *path: hsa05171* to COVID-19. Among the GO pathway terms, *GO:0035455* is notable for its links to “response to interferon-alpha”—a key cytokine used by the body to fight against viral infections and other immunological distresses.

### AUC of the algorithm

**Fig. 4:**
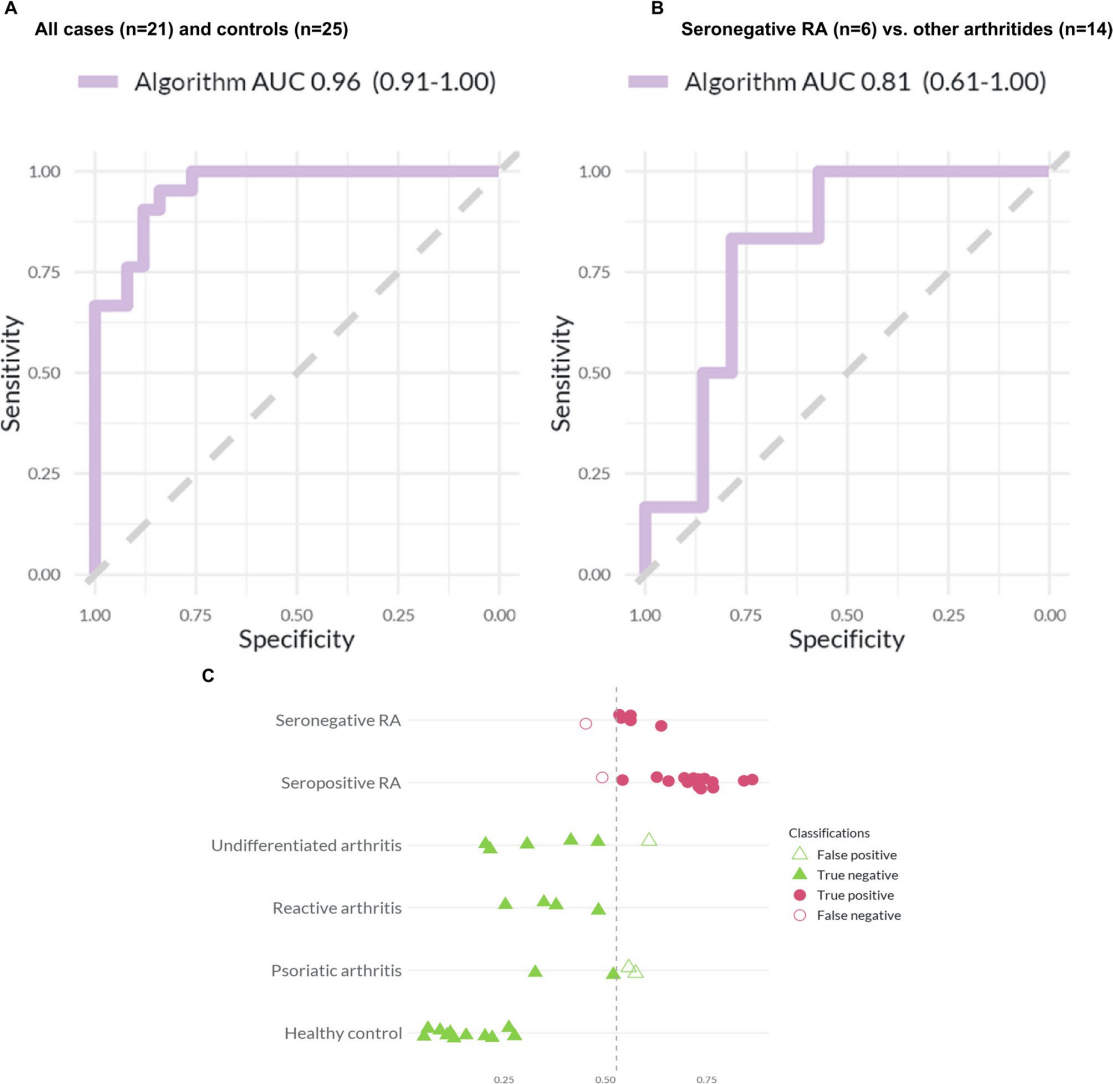
Prediction performance of the algorithm. Panels **A** and **B** show the area under the curve (AUC) for our classification algorithm in the holdout set. Panel **A** shows the comparison between all RA patients and all controls and Panel **B** is for seronegative RA versus other arthritides. Panel **C** shows the probability score per phenotypic group for all RA patients and all controls and in the holdout set. The x-axis displays the probability score for each individual, while the y-axis displays the different patient groups. The vertical stippled line indicates the optimal cutoff of 0.52; any score to the left of this cutoff is classified as non-RA and any score to the right as RA

In the holdout set, when comparing all RA patients (*n* = 21) with all controls (*n* = 25), our algorithm showed an AUC of 0.96 (95% CI: 0.91–1.0) (Fig. 4). When comparing patients with seronegative RA (*n* = 6) to those with other arthritides (*n* = 14), our algorithm showed an AUC of 0.81 (95% CI: 0.61–1.0) (Fig. 4).

In the holdout set, seropositive RA patients had a median algorithm probability score of 0.73 (interquartile range, IQR: 0.68–0.75), whereas seronegative RA patients had a median algorithm probability score of 0.55 (IQR: 0.54–0.56) (Supplementary Table 4). Patients with non-RA inflammatory arthritis had a median algorithm probability score of 0.40 (IQR: 0.31–0.51), whereas healthy individuals had a median algorithm probability score of 0.13 (IQR: 0.10–0.21). All participants in the lowest and highest quartiles were correctly classified in the entire holdout set (Supplementary Fig. 1). Similar results were obtained for the more clinically relevant scenario where the goal is to differentiate patients with seronegative RA from those with other arthritides (Supplementary Fig. 1).

### Sensitivity and specificity of our algorithm

We converted our algorithm’s probability score to a dichotomous value. In the training set, a cutoff of 0.52 was estimated with the Youden’s J statistic [32] to be optimal for this conversion, and was therefore used for all subsequent classifications. When applying this cutoff to analyses in the holdout set, our algorithm showed a sensitivity of 0.90 (95% CI: 0.70–0.99) and a specificity of 0.88 (95% CI: 0.69–0.97) when used to classify all RA patients (*n* = 21) from controls (*n* = 25) (Table 2). Our algorithm showed a sensitivity of 83% (95% CI: 0.36–1.00) and a specificity of 79% (95% CI: 0.49–0.95) when used to classify patients with seronegative RA (*n* = 6) and those with other arthritides (*n* = 14) (Table 2). For completeness, when used to classify patients with seropositive RA (*n* = 15) and those with other arthritides (*n* = 14) our algorithm showed a sensitivity of 93% (95% CI: 0.68–1.00) and a specificity of 79% (95% CI: 0.49–0.95) (Supplementary Table 5).

**Table 2:** Performance of our classification algorithm in the holdout set

	All cases (*n* = 21) and controls (*n* = 25)^2^				Seronegative RA (*n* = 6) vs. other arthritides (*n* = 14)^2^		
	Balanced Accuracy^3^	Sensitivity [95% CI]	Specificity [95% CI]		Balanced Accuracy	Sensitivity [95% CI]	Specificity [95% CI]
Algorithm^1^	0.89	0.90 [0.36–1.00]	0.88 [0.57–0.98]		0.81	0.83 [0.36–1.00]	0.79 [0.49–0.95]

^1^Our algorithm includes both serology (ACPA combined with RF) and DNAm features

^2^As the label implies, the “All cases and controls” holdout set includes all the cases and controls. The “Seronegative RA vs other arthritides” subset includes seronegative RA, undifferentiated arthritis, reactive arthritis, and psoriatic arthritis

^3^Balanced accuracy is a metric used to evaluate the performance of a classification algorithm when the data are imbalanced. It is the average of sensitivity and specificity

### Comparison of algorithm

In a post-hoc sensitivity analysis, we evaluated three alternate algorithm candidates: one with sex, age and serology as covariates, a second with sex, age, serology and DNAm as covariates, and a third with only DNAm as covariates (Supplementary Table 6). In the holdout set, our algorithm, which includes 391 DNAm features and serology as covariates, had the highest balanced accuracy across all the tested groups (full holdout, seronegative vs. other arthritides).

## Discussion

We developed a high-performance DNAm-based classification algorithm that leverages DNAm data from whole blood samples. It showed high accuracy in classifying RA patients, both seropositive and seronegative, from those with other types of arthritis. The results indicate a potential future clinical importance, especially in diagnosing seronegative patients who often experience delays in diagnosis and treatment initiation.

We are not aware of other DNAm-based tests that are specifically designed for identifying RA among other relevant arthritides. Anti-CCP and RF cannot, by definition, detect seronegative RA. One test capable of doing so — the “Augurex” test — measures both serology and the 14-3-3η protein in blood [34]. Although the sensitivity and specificity of the Augurex test for detecting RA were reported to be 0.73 and 0.88, respectively [35], its sensitivity for detecting seronegative RA was only 21%. By contrast, our algorithm achieved a sensitivity of 83% for detecting seronegative RA. If these findings are replicable in a clinical setting, our algorithm may help improve patient outcomes by diagnosing seronegative RA patients earlier.

Even though supplementary analyses showed a negligible difference in our algorithm’s performance when serology was excluded, we still chose to include serology to improve the ability to detect seropositive RA. While age, sex, and serology were incorporated as covariates when training the algorithm, only serology was retained in addition to the DNAm features. Our algorithm’s high sensitivity of 83% for classifying seronegative RA is largely due to the contribution of DNAm data. By contrast, our algorithm’s least effective differentiation was between psoriatic arthritis and RA. Furthermore, the limited sample size of psoriatic arthritis patients complicated the interpretation of these findings.

Patients with autoimmune diseases have been shown to display differences in DNAm in immune cells [36]. It is thus unsurprising to find a signal in leukocytes capable of detecting RA. Previous studies have also highlighted differences in DNAm between healthy controls and patients with RA [37, 38]. For instance, Ambatapuri et al. [37] identified differences in DNAm in leukocytes and myelocytes between untreated RA patients and healthy controls. They used this discrepancy to develop a DNAm-derived marker of systemic inflammation. This DNAm pattern was, however, not observed in RA patients who had undergone treatment. Since our study focused entirely on treatment-naïve RA patients, it is difficult to decipher whether the use of DMARDs could have affected our algorithm. Another study in which a genome-wide profiling of treatment-naive RA patients was conducted [39] identified significant differences in DNAm levels in lymphocytes early in the disease course. Feng et al. [38] also developed a DNAm-based algorithm that was capable of differentiating between healthy controls and RA patients with 100% accuracy. However, differentiating between healthy controls and RA patients does not reflect the clinical setting in which such a test would be useful. The algorithms mentioned above could instead be targeting general markers of inflammation.

The gene-enrichment and pathway analyses were limited in shedding light on the biological significance of the DNAm features included in our algorithm in relation to RA. Based on the FDR values, none of the reported pathways reached statistical significance, which raises concerns about the biological significance of these features and warrants further consideration. Nevertheless, the KEGG pathway analysis pointed to two pathways that are linked to viral infections: herpes simplex virus 1 and COVID-19. While viral infections are not recognized as direct risk factors for RA, they could potentially act as triggers. Earlier research has identified associations between RA and respiratory viral infections [40], as well as elevated titers of specific viruses [41, 42]. The results of our KEGG analysis also revealed pathways linked to autoimmune diseases, SLE and IBD, and the pathways also contained genes belonging to the major histocompatibility complex (MHC). From the top 10 GO pathway analysis, it is noteworthy that one of the pathways is linked to interferon alpha (IFN-alpha) response — a cytokine produced by the body in response to viral infections and inflammation.

This study has several strengths and limitations. One major strength is the use of relevant study populations, well-characterized samples, high-quality data across several clinical studies, and the large and diverse discovery set which enhances the robustness of the DNAm feature extraction. Another notable strength is the inclusion of other arthritides as controls, as opposed to only including healthy individuals. Although this complicates the task of accurate classification, it reflects real-world diagnostic challenges and increases the relevance of our algorithm. In addition, all RA cases and arthritides controls were treatment-naïve and were evaluated by rheumatologists. Limitations include the small sample size of the holdout set, the ethnically homogeneous sample populations, and the use of the rheumatologist’s final diagnosis as the endpoint in the analysis (the latter might introduce a subjective bias). Another limitation is the disparity in size between the discovery set and the modeling set, where the discovery set comprises a large number of samples while the modeling set is considerably smaller due to data availability and the need to mitigate technical artifacts. Furthermore, our algorithm was built using DNAm data from patient groups specific to certain studies, which could lead to the identification of batches rather than biologically relevant signals. However, the likelihood of bias due to batches is deemed low given that the blood samples were collected, processed, and stored using the same protocol and DNAm measurement of the modeling set was processed in a single batch. Another limitation is that the serology status (RF and ACPA) was not measured on the healthy controls in the modeling set and was thus set to negative serology status for analytical purposes. Although this is generally a safe assumption, it is important to note that RF and ACPA have been found to be positive in a small percentage of healthy individuals [43, 44]. Finally, another limitation is the use of neonatal DNAm data from NICU as a control phenotype for sepsis. Data from adult patients with severe infections were unfortunately not available, and as we aimed to capture as many different types of inflammatory diseases as possible to be able to gauge the specificity of our predictor for RA, we considered the NICU patients to add value as a comparator group for bacterial infection. Despite these shortcomings, our algorithm performed remarkably well in the holdout set. Addressing these limitations would require further validation in larger cohorts with a more ethnically diverse representation to ensure that the results are generalizable.

## Conclusions

In conclusion, our study, based on using extensive and robust datasets and validation in patients reflecting a clinically relevant setting, presents the development of a novel and promising diagnostic classification algorithm capable of accurately classifying seronegative and seropositive RA from other arthritides and healthy controls. Given the significant unmet need for a more timely and accurate identification of these patients, our algorithm may serve as a new tool for diagnosing RA, especially for the early identification of seronegative RA patients. Further research in larger cohorts is needed to confirm our findings.

## Supplementary Information

Below is the link to the electronic supplementary material.

Supplementary Material 1

## Data Availability

The DNAm data for the discovery set is publicly-available data from the GEO repositories with accession numbers GSE42861, GSE87648, GSE43976 and GSE155952. The DNAm data for the modelling set are not publicly available due to privacy and ethical restrictions.

## Abbreviations

ACPA: Anti-citrullinated peptide antibodies
ACR: American College of Rheumatology
ARCTIC: Aiming for Remission in RA
AUC: Area under the receiver-operating curve
CpG: Cytosine-phosphate-guanine
DMARDs: Disease-modifying antirheumatic drugs
DNAm: DNA methylation
EDTA: Ethylenediaminetetraacetic acid
EIMS: Epidemiological Investigation of Multiple Sclerosis
EIRA: Epidemiological Investigation of RA
EULAR: European Alliance of Associations for Rheumatology
EWAS: Epigenome-wide association studies
GEO: Gene Expression Omnibus
IBD: BIOM-Inflammatory Bowel Disease Biomarkers Programme
IFN: Alpha-interferon alpha
IQR: Interquartile range
MHC: Major histocompatibility complex
NICU: Neonatal Intensive Care Unit Study
NOR: VEAC-Norwegian Very Early Arthritis Clinic
PREDCOV: Predict COVID
RA: Rheumatoid arthritis
RF: Rheumatoid factor
SLE: Systemic lupus erythematosus
