# Supplementary material for: A DNA methylation-based algorithm for diagnosing rheumatoid arthritis
Source: Arthritis Res Ther. 2025 Oct 17;27:192. doi: 10.1186/s13075-025-03649-x (PMC12532955; doi:10.1186/s13075-025-03649-x)
Supplement: Supplementary file 1 — Supplementary Material 1 [file 13075_2025_3649_MOESM1_ESM.docx]

# Supplementary Material

*“A DNA methylation-based algorithm for diagnosing rheumatoid arthritis“*

## Supplementary Tables

### **Supplementary Table 1:** Top 10 KEGG pathways

| **KEGG pathway ID** | **Description** | **N** | **DE** | **P.DE** | **FDR** | **SigGenesInSet** |
| --- | --- | --- | --- | --- | --- | --- |
| [path:hsa05168](https://www.genome.jp/dbget-bin/www_bget?path:hsa05168) | Herpes simplex virus 1 infection | 506.00 | 14.00 | 0.01 | 1.00 | [*ZNF605*](https://www.genecards.org/cgi-bin/carddisp.pl?gene=ZNF605)*,* [*ZNF268*](https://www.genecards.org/cgi-bin/carddisp.pl?gene=ZNF268)*,* [*ZNF595*](https://www.genecards.org/cgi-bin/carddisp.pl?gene=ZNF595)*,* [*ZNF584*](https://www.genecards.org/cgi-bin/carddisp.pl?gene=ZNF584)*,* [*TAB2*](https://www.genecards.org/cgi-bin/carddisp.pl?gene=TAB2)*,* [*ZNF718*](https://www.genecards.org/cgi-bin/carddisp.pl?gene=ZNF718)*,* [*HLA*](https://www.genecards.org/cgi-bin/carddisp.pl?gene=HLA)*,* [*DQB1*](https://www.genecards.org/cgi-bin/carddisp.pl?gene=DQB1)*,* [*ZNF517*](https://www.genecards.org/cgi-bin/carddisp.pl?gene=ZNF517)*,* [*ZNF761*](https://www.genecards.org/cgi-bin/carddisp.pl?gene=ZNF761)*,* [*ZNF324B*](https://www.genecards.org/cgi-bin/carddisp.pl?gene=ZNF324B)*,* [*EIF2AK2*](https://www.genecards.org/cgi-bin/carddisp.pl?gene=EIF2AK2)*,* [*ZNF10*](https://www.genecards.org/cgi-bin/carddisp.pl?gene=ZNF10)*,* [*ZNF140*](https://www.genecards.org/cgi-bin/carddisp.pl?gene=ZNF140)*,* [*ZNF141*](https://www.genecards.org/cgi-bin/carddisp.pl?gene=ZNF141)*,* [*ZNF251*](https://www.genecards.org/cgi-bin/carddisp.pl?gene=ZNF251) |
| [path:hsa00564](https://www.genome.jp/dbget-bin/www_bget?path:hsa00564) | Glycerophospholipid metabolism | 98.00 | 5.00 | 0.03 | 1.00 | [*AGPAT1*](https://www.genecards.org/cgi-bin/carddisp.pl?gene=AGPAT1)*,* [*GPAT4*](https://www.genecards.org/cgi-bin/carddisp.pl?gene=GPAT4)*,* [*PTDSS2*](https://www.genecards.org/cgi-bin/carddisp.pl?gene=PTDSS2)*,* [*DGKD*](https://www.genecards.org/cgi-bin/carddisp.pl?gene=DGKD)*,* [*PLA2G4C*](https://www.genecards.org/cgi-bin/carddisp.pl?gene=PLA2G4C) |
| [path:hsa05322](https://www.genome.jp/dbget-bin/www_bget?path:hsa05322) | Systemic lupus erythematosus | 120.00 | 4.00 | 0.04 | 1.00 | [*H2AC8*](https://www.genecards.org/cgi-bin/carddisp.pl?gene=H2AC8)*,* [*HLA*](https://www.genecards.org/cgi-bin/carddisp.pl?gene=HLA)*,* [*DQB1*](https://www.genecards.org/cgi-bin/carddisp.pl?gene=DQB1)*,* [*C8A*](https://www.genecards.org/cgi-bin/carddisp.pl?gene=C8A)*,* [*H3C11*](https://www.genecards.org/cgi-bin/carddisp.pl?gene=H3C11)*,* [*H4C13*](https://www.genecards.org/cgi-bin/carddisp.pl?gene=H4C13) |
| [path:hsa00531](https://www.genome.jp/dbget-bin/www_bget?path:hsa00531) | Glycosaminoglycan degradation | 19.00 | 2.00 | 0.05 | 1.00 | [*GNS*](https://www.genecards.org/cgi-bin/carddisp.pl?gene=GNS)*,* [*GUSB*](https://www.genecards.org/cgi-bin/carddisp.pl?gene=GUSB) |
| [path:hsa00061](https://www.genome.jp/dbget-bin/www_bget?path:hsa00061) | Fatty acid biosynthesis | 17.00 | 2.00 | 0.05 | 1.00 | [*ACSF3*](https://www.genecards.org/cgi-bin/carddisp.pl?gene=ACSF3)*,* [*ACSL1*](https://www.genecards.org/cgi-bin/carddisp.pl?gene=ACSL1) |
| [path:hsa04217](https://www.genome.jp/dbget-bin/www_bget?path:hsa04217) | Necroptosis | 157.00 | 5.00 | 0.06 | 1.00 | [*PARP1*](https://www.genecards.org/cgi-bin/carddisp.pl?gene=PARP1)*,* [*CHMP2A*](https://www.genecards.org/cgi-bin/carddisp.pl?gene=CHMP2A)*,* [*H2AC8*](https://www.genecards.org/cgi-bin/carddisp.pl?gene=H2AC8)*,* [*EIF2AK2*](https://www.genecards.org/cgi-bin/carddisp.pl?gene=EIF2AK2)*,* [*PLA2G4C*](https://www.genecards.org/cgi-bin/carddisp.pl?gene=PLA2G4C) |
| [path:hsa05171](https://www.genome.jp/dbget-bin/www_bget?path:hsa05171) | Coronavirus disease - COVID-19 | 231.00 | 6.00 | 0.06 | 1.00 | [*TAB2*](https://www.genecards.org/cgi-bin/carddisp.pl?gene=TAB2)*,* [*EIF2AK2*](https://www.genecards.org/cgi-bin/carddisp.pl?gene=EIF2AK2)*,* [*RPL8*](https://www.genecards.org/cgi-bin/carddisp.pl?gene=RPL8)*,* [*RPL19*](https://www.genecards.org/cgi-bin/carddisp.pl?gene=RPL19)*,* [*RPS5*](https://www.genecards.org/cgi-bin/carddisp.pl?gene=RPS5)*,* [*C8A*](https://www.genecards.org/cgi-bin/carddisp.pl?gene=C8A) |
| [path:hsa05321](https://www.genome.jp/dbget-bin/www_bget?path:hsa05321) | Inflammatory bowel disease | 63.00 | 3.00 | 0.08 | 1.00 | [*HLA*](https://www.genecards.org/cgi-bin/carddisp.pl?gene=HLA)*,* [*DQB1*](https://www.genecards.org/cgi-bin/carddisp.pl?gene=DQB1)*,* [*SMAD3*](https://www.genecards.org/cgi-bin/carddisp.pl?gene=SMAD3)*,* [*NFATC1*](https://www.genecards.org/cgi-bin/carddisp.pl?gene=NFATC1) |
| [path:hsa04130](https://www.genome.jp/dbget-bin/www_bget?path:hsa04130) | SNARE interactions in vesicular transport | 32.00 | 2.00 | 0.08 | 1.00 | [*VTI1B*](https://www.genecards.org/cgi-bin/carddisp.pl?gene=VTI1B)*,* [*BET1L*](https://www.genecards.org/cgi-bin/carddisp.pl?gene=BET1L) |
| [path:hsa00561](https://www.genome.jp/dbget-bin/www_bget?path:hsa00561) | Glycerolipid metabolism | 61.00 | 3.00 | 0.10 | 1.00 | [*AGPAT1*](https://www.genecards.org/cgi-bin/carddisp.pl?gene=AGPAT1)*,* [*GPAT4*](https://www.genecards.org/cgi-bin/carddisp.pl?gene=GPAT4)*,* [*DGKD*](https://www.genecards.org/cgi-bin/carddisp.pl?gene=DGKD) |

Shown here are the top 10 most significantly enriched KEGG pathways, sorted by the ‘p-value adjusted for over-representation on array’ (denoted here as “P.DE”). “N” is the number of genes in the pathway, “DE” is the number of genes found to be differentially methylated, “P.DE” is the p-value adjusted for over-representation on array, “FDR” is the false discovery rate calculated using the Benjamini-Hochberg procedure, and “SigGenesInSet” is the list of significant differentially methylated genes within that pathway.

### **Supplementary Table 2:** Top 10 GO pathways

| **GO pathway ID** | **ONTOLOGY** | **TERM** | **N** | **DE** | **P.DE** | **FDR** |
| --- | --- | --- | --- | --- | --- | --- |
| [GO:2000825](https://www.ebi.ac.uk/QuickGO/term/GO:2000825) | BP | Positive regulation of androgen receptor activity | 3.00 | 2.00 | 0.00 | 1.00 |
| [GO:0019843](https://www.ebi.ac.uk/QuickGO/term/GO:0019843) | MF | rRNA binding | 67.00 | 5.00 | 0.00 | 1.00 |
| [GO:0140214](https://www.ebi.ac.uk/QuickGO/term/GO:0140214) | BP | Positive regulation of long-chain fatty acid import into cell | 3.00 | 2.00 | 0.00 | 1.00 |
| [GO:0035455](https://www.ebi.ac.uk/QuickGO/term/GO:0035455) | BP | Response to interferon-alpha | 20.00 | 3.00 | 0.00 | 1.00 |
| [GO:0031397](https://www.ebi.ac.uk/QuickGO/term/GO:0031397) | BP | Negative regulation of protein ubiquitination | 83.00 | 6.00 | 0.00 | 1.00 |
| [GO:0006654](https://www.ebi.ac.uk/QuickGO/term/GO:0006654) | BP | Phosphatidic acid biosynthetic process | 32.00 | 4.00 | 0.00 | 1.00 |
| [GO:0045017](https://www.ebi.ac.uk/QuickGO/term/GO:0045017) | BP | Glycerolipid biosynthetic process | 249.00 | 11.00 | 0.00 | 1.00 |
| [GO:0046474](https://www.ebi.ac.uk/QuickGO/term/GO:0046474) | BP | Glycerophospholipid biosynthetic process | 209.00 | 10.00 | 0.00 | 1.00 |
| [GO:1903321](https://www.ebi.ac.uk/QuickGO/term/GO:1903321) | BP | Negative regulation of protein modification by small protein conjugation or removal | 94.00 | 6.00 | 0.00 | 1.00 |
| [GO:0046473](https://www.ebi.ac.uk/QuickGO/term/GO:0046473) | BP | Phosphatidic acid metabolic process | 36.00 | 4.00 | 0.01 | 1.00 |

Shown here are the top 10 most significantly enriched GO pathways, sorted by the ‘p-value adjusted for over-representation on array’ (P.DE). As in Supplementary Table 1, N is the number of genes in the pathway, DE is the number of genes found to be differentially methylated, P.DE is the p-value adjusted for over-representation on array, and FDR is the false discovery rates calculated using the Benjamini-Hochberg procedure. ONTOLOGY is either BP - biological process, CC - cellular component, or MF - molecular function.

###

### **Supplementary Table 3:** Synopsis of the top 20 CpGs and associated genes

| **CpG ID** | **Chromosome** | **Gene ID** | **Gene name** | **Comments^1^** |
| --- | --- | --- | --- | --- |
| [cg02196834](http://www.ewascatalog.org/?query=cg02196834) | [chr6](http://www.ensembl.org/Homo_sapiens/Location/Chromosome?r=6) | NA |  | NA |
| [cg20843080](http://www.ewascatalog.org/?query=cg20843080) | [chr12](http://www.ensembl.org/Homo_sapiens/Location/Chromosome?r=12) | [*DYNLL1*](https://www.genecards.org/cgi-bin/carddisp.pl?gene=DYNLL1) | Dynein Light Chain LC8-Type 1 | This gene belongs to a family of large complexes that are involved in intracellular transport and motility. Mutations in *DYNLL1* have been linked to different cancers of the epithelial cells, including prostate carcinoma in situ and breast adenoid cystic carcinoma. Gene Ontology (GO) annotations related to this gene include protein homodimerization activity and protein domain specific binding. |
| [cg22174356](http://www.ewascatalog.org/?query=cg22174356) | [chr13](http://www.ensembl.org/Homo_sapiens/Location/Chromosome?r=13) | [*ZMYM2*](https://www.genecards.org/cgi-bin/carddisp.pl?gene=ZMYM2) | Zinc Finger MYM-Type Containing 2 | This gene codes for a zinc finger protein believed to function as a transcription factor. The protein might be involved in a BHC histone deacetylase complex. Translocation of this gene with fibroblast growth factor receptor-1 (*FGFR1*) may be associated with the onset of stem cell leukemia lymphoma syndrome (SCLL). |
| [cg12876900](http://www.ewascatalog.org/?query=cg12876900) | [chr11](http://www.ensembl.org/Homo_sapiens/Location/Chromosome?r=11) | [*IFITM3*](https://www.genecards.org/cgi-bin/carddisp.pl?gene=IFITM3) | Interferon Induced Transmembrane Protein 3 | This gene encodes a protein belonging to the interferon-induced transmembrane (IFITM) family. The protein product of this gene inhibits the entry of various viral pathogens into cells, including influenza A virus, Ebola virus, and Sars-CoV-2. Conditions associated with *IFITM3* include influenza. Among its related pathways are cytokine signaling in the immune system. |
| [cg08669718](http://www.ewascatalog.org/?query=cg08669718) | [chr4](http://www.ensembl.org/Homo_sapiens/Location/Chromosome?r=4) | [*ZNF718*](https://www.genecards.org/cgi-bin/carddisp.pl?gene=ZNF718) | Zinc Finger Protein 718 | The protein product of this gene is predicted to be involved in regulation of transcription, specifically in enabling DNA-binding transcription factor activity, RNA polymerase II-specific and RNA polymerase II cis-regulatory region sequence-specific DNA binding activity. |
| [cg14402591](http://www.ewascatalog.org/?query=cg14402591) | [chr19](http://www.ensembl.org/Homo_sapiens/Location/Chromosome?r=19) | [*SEMA6B*](https://www.genecards.org/cgi-bin/carddisp.pl?gene=SEMA6B) | Semaphorin 6B | This gene produces a protein that is part of the semaphorin family, which is distinguished by the presence of a conserved semaphorin (sema) domain. Semaphorins are crucial for axon guidance and the development of both the peripheral and central nervous systems. *SEMA6B* has been linked to conditions such as epilepsy and diffuse glomerulonephritis. Gene Ontology (GO) annotations connected to this gene involve signaling receptor function and semaphorin receptor interaction. |
| [cg20000994](http://www.ewascatalog.org/?query=cg20000994) | [chr6](http://www.ensembl.org/Homo_sapiens/Location/Chromosome?r=6) | [*ZBTB12*](https://www.genecards.org/cgi-bin/carddisp.pl?gene=ZBTB12) | Zinc Finger And BTB Domain Containing 12 | This gene produces a protein that is anticipated to participate in double-stranded DNA binding activity with sequence specificity and in the regulation of transcription mediated by RNA polymerase II. Conditions linked to *ZBTB12* involve benign immune system organ neoplasms and thymus lipoma. |
| [cg26035007](http://www.ewascatalog.org/?query=cg26035007) | [chr4](http://www.ensembl.org/Homo_sapiens/Location/Chromosome?r=4) | NA | NA | NA |
| [cg09591303](http://www.ewascatalog.org/?query=cg09591303) | [chr13](http://www.ensembl.org/Homo_sapiens/Location/Chromosome?r=13) | [*TFDP1*](https://www.genecards.org/cgi-bin/carddisp.pl?gene=TFDP1) | Transcription Factor Dp-1 | This gene encodes a protein belonging to a group of transcription factors that form heterodimers with E2F proteins, enhancing their DNA-binding capacity and promoting transcription from E2F target genes. [TFDP1](https://www.genecards.org/cgi-bin/carddisp.pl?gene=TFDP1) is a part of this complex, regulating the transcriptional activity of numerous genes involved in the cell cycle progression from G1 to S phase. Pseudogenes of this gene are located on chromosomes 1, 15, and X. This gene is associated with diseases such as colorectal cancer and hepatocellular carcinoma. Some of the related pathways include aberrant regulation of the mitotic G1/S transition in cancer due to RB1 defects and the intrinsic pathway for apoptosis. Additionally, GO annotations connected to this gene involve DNA-binding transcription factor function and transcription coactivator activity. |
| [cg07777224](http://www.ewascatalog.org/?query=cg07777224) | [chr19](http://www.ensembl.org/Homo_sapiens/Location/Chromosome?r=19) | [*ZNF584*](https://www.genecards.org/cgi-bin/carddisp.pl?gene=ZNF584) | Zinc Finger Protein 584 | The protein encoded by this gene is anticipated to possess DNA-binding transcription factor activity specific to RNA polymerase II and to bind RNA polymerase II cis-regulatory region sequence-specific DNA. *ZNF584* is associated with conditions such as intellectual developmental disorder with cardiac arrhythmia. GO annotations connected to this gene involve nucleic acid binding. |
| [cg03175049](http://www.ewascatalog.org/?query=cg03175049) | [chr19](http://www.ensembl.org/Homo_sapiens/Location/Chromosome?r=19) | [*CHMP2A*](https://www.genecards.org/cgi-bin/carddisp.pl?gene=CHMP2A) | Charged Multivesicular Body Protein 2A | This gene encodes a member of the chromatin-modifying protein/charged multivesicular body protein (CHMP) family. These proteins are part of ESCRT-III (endosomal sorting complex required for transport III), a complex that plays a role in surface receptor protein degradation and endocytic multivesicular body (MVB) formation. Some CHMP proteins exhibit both nuclear and cytoplasmic/vesicular localizations, such as CHMP1A (MIM 164010), which is necessary for both MVB formation and cell cycle regulation. Diseases linked to *CHMP2A* include breast adenocarcinoma and frontotemporal dementia and/or amyotrophic lateral sclerosis 7. Related pathways to this gene involve early SARS-CoV-2 infection events and the HIV life cycle. GO annotations associated with this gene include protein domain-specific binding and phosphatidylcholine binding. |
| [cg21883757](http://www.ewascatalog.org/?query=cg21883757) | [chr22](http://www.ensembl.org/Homo_sapiens/Location/Chromosome?r=22) | NA | NA | NA |
| [cg14369970](http://www.ewascatalog.org/?query=cg14369970) | [chr1](http://www.ensembl.org/Homo_sapiens/Location/Chromosome?r=1) | [*PGBD2*](https://www.genecards.org/cgi-bin/carddisp.pl?gene=PGBD2) | PiggyBac Transposable Element Derived 2 | This gene is part of the piggyBac transposable element-derived (PGBD) subfamily. The piggyBac family of proteins, identified in various animals, are transposases similar to the transposase of the original piggyBac transposon found in the moth, *Trichoplusia ni*. This family also encompasses genes in several genomes, including the human genome, which appear to have originated from piggyBac transposons. PGBD proteins seem to be unique, with no evident connection to other transposases or known protein families. The precise function of this gene remains unclear. Two transcript variants encoding distinct isoforms have been identified for this gene. Diseases linked to *PGBD2* include Cockayne Syndrome B and Wolf-Hirschhorn Syndrome. |
| [cg21149260](http://www.ewascatalog.org/?query=cg21149260) | [chr1](http://www.ensembl.org/Homo_sapiens/Location/Chromosome?r=1) | [*ZNF672*](https://www.genecards.org/cgi-bin/carddisp.pl?gene=ZNF672) | Zinc Finger Protein 672 | The protein encoded by this gene is expected to have DNA-binding transcription factor activity specific to RNA polymerase II and sequence-specific DNA binding capabilities. It is predicted to participate in the regulation of transcription by RNA polymerase II. *ZNF672* has been associated with Wolf-Hirschhorn Syndrome. GO annotations connected to this gene involve nucleic acid binding. |
| [cg08871354](http://www.ewascatalog.org/?query=cg08871354) | [chr16](http://www.ensembl.org/Homo_sapiens/Location/Chromosome?r=1) | [*GINS2*](https://www.genecards.org/cgi-bin/carddisp.pl?gene=GINS2) | GINS Complex Subunit 2 | The GINS complex in yeast is a heterotetrameric structure consisting of Sld5 (GINS4; MIM 610611), Psf1 (GINS1; MIM 610608), Psf2, and Psf3 (GINS3; MIM 610610). The formation of this complex is crucial for initiating DNA replication in both yeast and Xenopus egg extracts. Diseases linked to *GINS2* include colon small cell carcinoma and Meier-Gorlin Syndrome 5. Some related pathways involve cell cycle mitotic processes and the regulation of activated PAK-2p34 degradation by the proteasome. GO annotations connected to this gene include 3'-5' DNA helicase activity. |
| [cg27632050](http://www.ewascatalog.org/?query=cg27632050) | [chr13](http://www.ensembl.org/Homo_sapiens/Location/Chromosome?r=13) | [*RASA3*](https://www.genecards.org/cgi-bin/carddisp.pl?gene=RASA3) | RAS P21 Protein Activator 3 | This gene codes for a protein that binds to inositol 1,3,4,5-tetrakisphosphate and enhances the GTPase activity of Ras p21. The protein functions as a negative regulator of the Ras signaling pathway and is localized to the cell membrane via a pleckstrin homology (PH) domain in the C-terminal region. Diseases associated with *RASA3* include paralytic poliomyelitis and poliomyelitis. Related pathways involve the RAF/MAP kinase cascade and regulation of activated PAK-2p34 degradation by the proteasome. GO annotations connected to this gene include GTPase activator activity and calcium-release channel activity. |
| [cg14830118](http://www.ewascatalog.org/?query=cg14830118) | [chr17](http://www.ensembl.org/Homo_sapiens/Location/Chromosome?r=1) | [*FAM18B*](https://www.genecards.org/cgi-bin/carddisp.pl?gene=FAM18B) | Trans-Golgi Network Vesicle Protein 23 Homolog B | The protein encoded by this gene is predicted to participate in protein secretion and vesicle-mediated transport. It is anticipated to be an integral component of the membrane and specifically of the Golgi membrane. Diseases associated with *TVP23B* include Charcot-Marie-Tooth Disease, Demyelinating, Type 1A. |
| [cg14164252](http://www.ewascatalog.org/?query=cg14164252) | [chr22](http://www.ensembl.org/Homo_sapiens/Location/Chromosome?r=22) | [*ARSA*](https://www.genecards.org/cgi-bin/carddisp.pl?gene=ARSA) | Arylsulfatase A | The protein encoded by this gene hydrolyzes cerebroside sulfate to cerebroside and sulfate. Mutations in this gene result in metachromatic leukodystrophy (MLD), a progressive demyelination disorder that causes various neurological symptoms and ultimately leads to death. Diseases associated with *ARSA* include metachromatic leukodystrophy and its juvenile form. Related pathways involve the innate immune system and protein metabolism. GO annotations connected to this gene include calcium ion binding and arylsulfatase activity. |
| [cg26329816](http://www.ewascatalog.org/?query=cg26329816) | [chr1](http://www.ensembl.org/Homo_sapiens/Location/Chromosome?r=1) | NA |  | NA |
| [cg18792041](http://www.ewascatalog.org/?query=cg18792041) | [chr11](http://www.ensembl.org/Homo_sapiens/Location/Chromosome?r=11) | [*ANO9*](https://www.genecards.org/cgi-bin/carddisp.pl?gene=ANO9) | Anoctamin 9 | The protein encoded by this gene belongs to the TMEM16 (anoctamin) family, which includes proteins that form integral membrane calcium-activated chloride channels. The exact function of the encoded protein remains to be determined, although it may possess channel-forming abilities and phospholipid scramblase activity. This gene has been found to be upregulated in stage II and III colorectal cancers. Moreover, diseases associated with ANO9 include g nathodiaphyseal dysplasia and Scott syndrome. Related pathways involve SARS-CoV-2 infection and ion channel transport. GO annotations connected to this gene include intracellular calcium-activated chloride channel activity and phospholipid scramblase activity. |

^1^ Information on the genes was collated from various sources, including NCBI Gene (<https://www.ncbi.nlm.nih.gov/gene> ), Gene Cards (<https://www.genecards.org/>), and OMIM (<https://www.omim.org/>).

###

### **Supplementary Table 4:** Median algorithm probability score by group

| **Group** | **Median** | **IQR** | **Q1** | **Q3** |
| --- | --- | --- | --- | --- |
| Healthy control | 0.13 | 0.11 | 0.10 | 0.21 |
| Other arthritides | 0.40 | 0.20 | 0.31 | 0.51 |
| Seronegative RA | 0.55 | 0.03 | 0.54 | 0.56 |
| Seropositive RA | 0.73 | 0.08 | 0.68 | 0.75 |

Shown here is the median algorithm probability score (Median) across diagnostic groups (first column) in the holdout set (n = 46), with the interquartile range (IQR) and the first and third quartiles (Q1 and Q3).

###

### **Supplementary Table 5:** Performance of our classification algorithm in the holdout set comparing seropositive RA vs other arthritides

|  | Seropositive RA (n=15) vs. other arthritides (n=14)^2^ | | |
| --- | --- | --- | --- |
|  | Balanced Accuracy | Sensitivity [95% CI] | Specificity [95% CI] |
| Algorithm^1^ | 0.86 | 0.93 [0.68-1.00] | 0.79 [0.49-0.95] |

^1^Our algorithm includes both serology (ACPA combined with RF) and DNAm features.

^2^The “Seropositive RA vs other arthritides” subset includes seropositive RA, undifferentiated arthritis, reactive arthritis, and psoriatic arthritis.

^3^Balanced accuracy is a metric used to evaluate the performance of a classification algorithm when the data are imbalanced. It is the average of sensitivity and specificity.

###

### **Supplementary Table 6:** Our classification algorithm and additional classification algorithm candidates tested in the holdout set

|  | **All cases (n=21) and**  **controls (n=25)** | | |  | **Seronegative RA (n=6) vs. other arthritides (n=14)** | | |
| --- | --- | --- | --- | --- | --- | --- | --- |
| **Candidate** | Balanced Accuracy | Sensitivity [95% CI] | Specificity [95% CI] |  | Balanced Accuracy | Sensitivity [95% CI] | Specificity[95% CI] |
| Our classification algorithm (Serology + DNAm) | 0.89 | 0.90  [0.70-0.99] | 0.88  [0.69-0.97] |  | 0.81 | 0.83  [0.36-1.00] | 0.79  [0.49-0.95] |
| Alternative classification algorithm #1 (Age + Sex + Serology) | 0.84 | 0.76  [0.53-0.92] | 0.92  [0.74-0.99] |  | 0.58 | 0.17  [0.00-0.64] | 1.00  [0.77-1.00] |
| Alternative classification algorithm #2 (Age + Sex + Serology + DNAm) | 0.86 | 0.95  [0.76-1.00] | 0.76  [0.55-0.91] |  | 0.70 | 0.83  [0.36-1.00] | 0.57  [0.29-0.82] |
| Alternative classification algorithm #3 (DNAm only) | 0.81 | 0.86  [0.64-0.97] | 0.76  [0.55-0.91] |  | 0.70 | 0.83  [0.36-1.00] | 0.57  [0.29-0.82] |

Shown here is the performance of our classification algorithm (first row) compared to that of three alternative classification algorithms. The left column represents the result of comparing all RA patients to all controls, and the right column the comparison between seronegative RA and other arthritides (the latter category includes psoriatic arthritis, reactive arthritis, and undifferentiated arthritis).

## Supplementary Figures

### **Supplementary Figure 1:** Prediction quartiles for our algorithm’s probability score

| 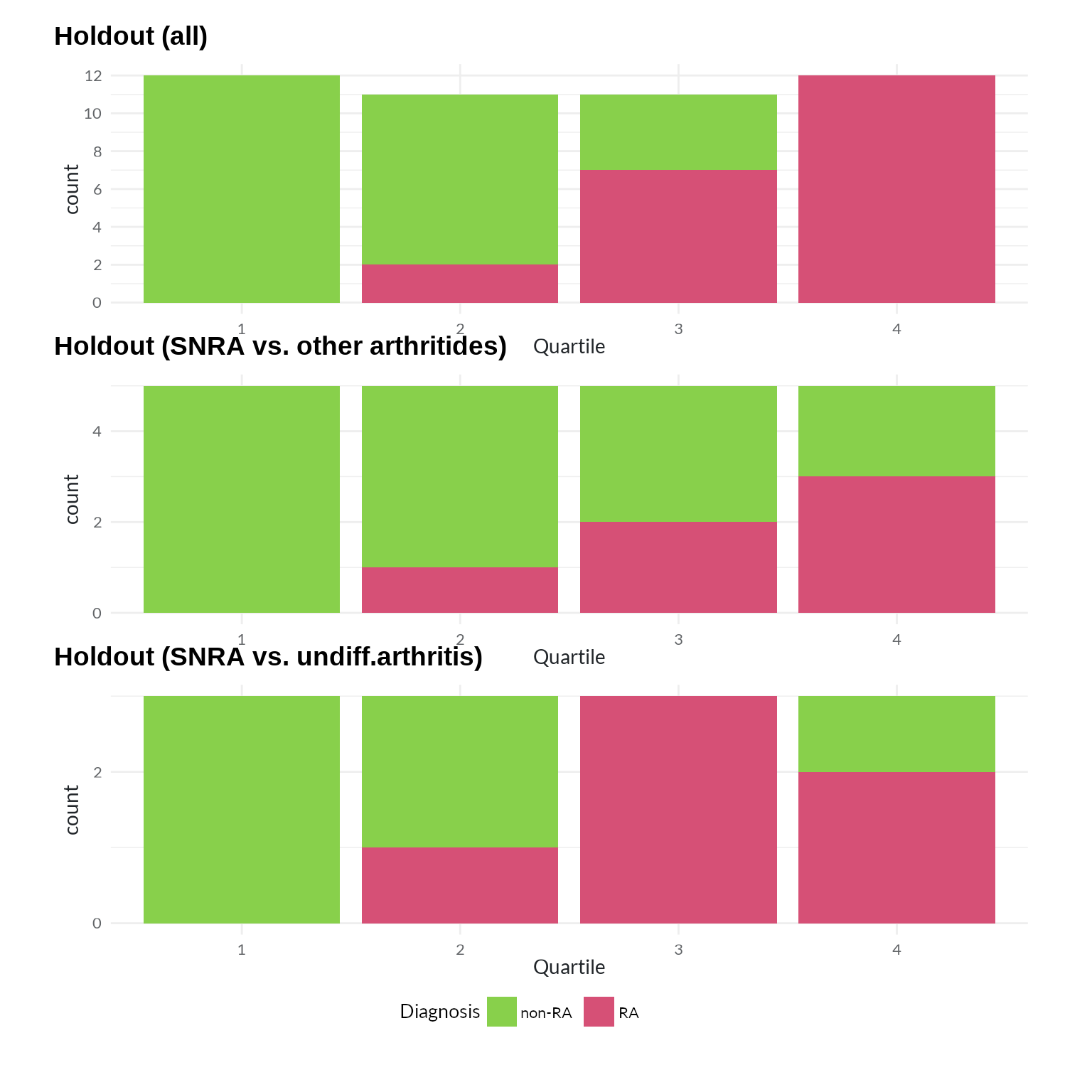 |
| --- |
| 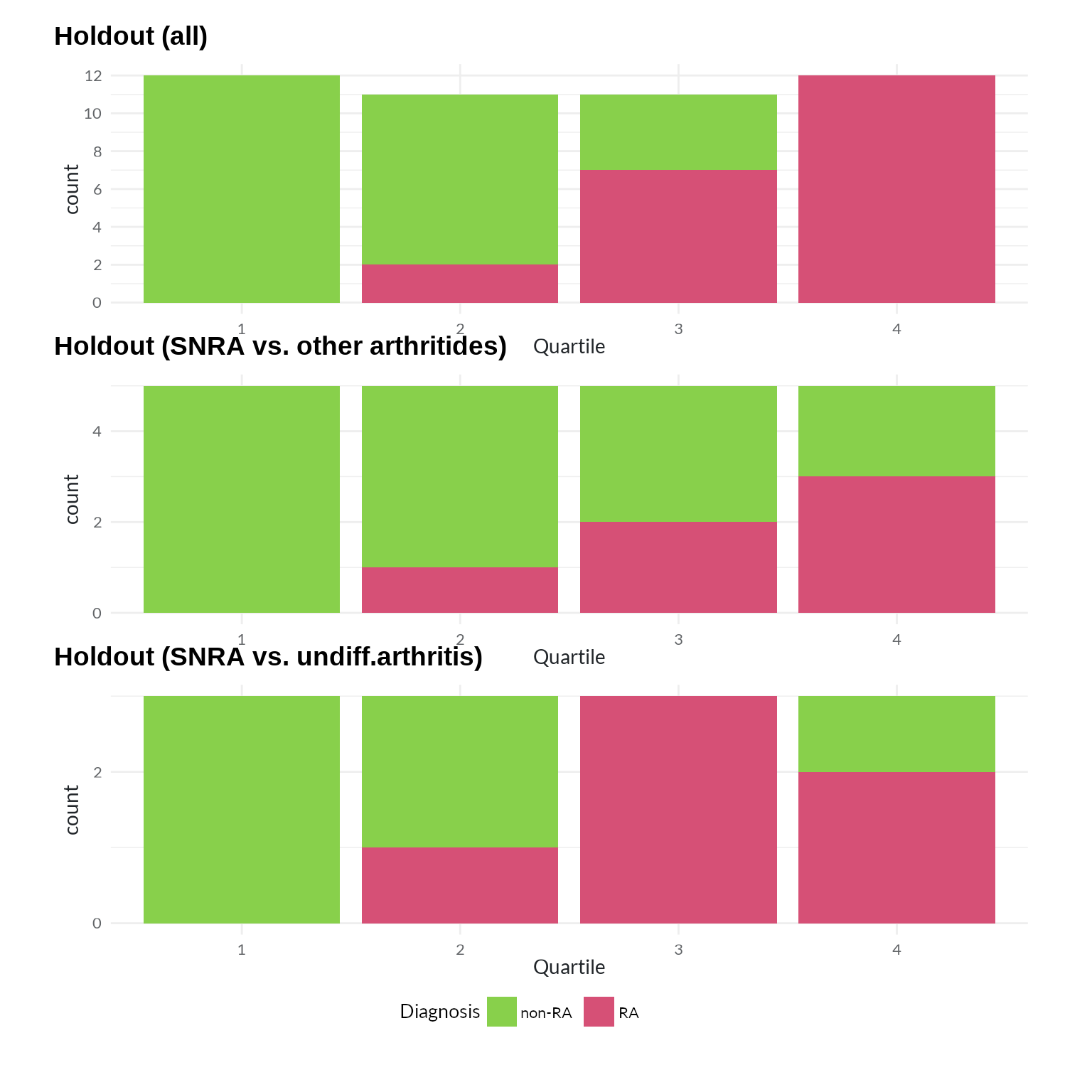 |

The figure shows our algorithm’s probability scores separated into four intervals (1, 2, 3, 4) using the quartiles 25%, 50% and 75% for the holdout set and subgroups of the holdout set. The y-axis shows the number of patients. The fill color signifies the diagnosis of each patient, with non-RA (green) indicating that the patient does not have RA, and RA (pink) indicating that the patient has RA.

Abbreviations: SNRA=seronegative RA.

## Supplementary Text

### Supplementary Text 1: Bioinformatics methods

#### Processing of DNAm data

To minimize the risk of systematic bias, we applied the same analytic pipeline to all the datasets (Figure 1 in the main text). Briefly, a standard pipeline in the ***R*** package {minfi} [(1)](https://sciwheel.com/work/citation?ids=488308&pre=&suf=&sa=0) was applied to extract the raw signal intensity data from the IDAT files. Background correction and normalization were performed using the default settings of the preprocessNoob function in {minfi}. For the discovery set, we combined the whole-blood DNAm datasets from GEO. The DNAm data from NICU was generated using the Illumina Human Methylation EPIC BeadChip, V1 (Illumina Inc., San Diego, USA), whereas those from EIRA, EIMS and IBD-BIOM were generated using Illumina Human Methylation 450K BeadChip. We harmonized the EPIC and 450K DNAm data by excluding the EPIC-only CpGs using convertArray from {minfi} and imputing the 32,786 missing 450K-only CpGs using their mean values from the discovery set.

#### Feature extraction

Feature extraction was performed in the discovery set (450K-based) to identify candidate CpG sites associated with RA. The resulting candidate features were then used to guide the training of classification algorithms on the modeling set (EPIC-based). We used three feature selection strategies: i) stability selection, ii) epigenome-wide association study (EWAS), and iii) elastic net classifier. For stability selection, we trained 100 classification algorithms and identified which of the features were consistently retained based on their selection probability. Combining the glmnet.lasso function with stabsel from the {stabs} [(2)](https://sciwheel.com/work/citation?ids=3491929&pre=&suf=&sa=0) and {glmnet} [(3)](https://sciwheel.com/work/citation?ids=171561&pre=&suf=&sa=0) packages, we employed the sampling scheme of Shah & Samworth [(4)](https://sciwheel.com/work/citation?ids=1509931&pre=&suf=&sa=0) based on 50 complementary pairs, setting the probability cutoff to 0.6 and allowing up to 350 selected variables per classification algorithm.

We performed an EWAS using the cpg.assoc function in {CpGassoc} [(5)](https://sciwheel.com/work/citation?ids=2040261&pre=&suf=&sa=0). An optimized classification algorithm was then trained using elastic net, with non-zero coefficients selected as feature candidates. The cv.biglasso function from the {biglasso} package [(6)](https://sciwheel.com/work/citation?ids=14859174&pre=&suf=&sa=0) was used for *k*-fold cross-validation training of penalized logistic regression to identify the optimal tuning parameters (alpha and lambda values) and the best performing classification algorithm.

#### Algorithm training

To identify the best-performing classification algorithm, we conducted a grid search using the following variables: (i) inclusion/exclusion of serology (RF and ACPA), (ii) the number of predictors used, and (iii) the candidate CpGs selected from the above-described feature extraction process.

For each permutation, we trained an elastic net binary classifier on the training set, with the final diagnosis assigned by the rheumatologist as the outcome. Elastic net is a regularization method that combines the L1 and L2 penalties to achieve an optimal balance between sparsity and correlation of features. We used the cv.biglasso function from the {biglasso} package. Through a 9-fold cross-validation, we estimated the optimal regularization parameter, lambda. To find the optimal value for alpha, we repeated the process using 10 different values of alpha, which controls the balance between the L1 and L2 penalties. The area under the curve (AUC) was used as a metric to evaluate the performance of the classification algorithm.

We used the majorization-minimization ("MM") option to calculate the update step in the optimization process. By default, all independent variables were assigned a penalty factor of 1, except for the candidate features, which were assigned a penalty factor of 0.5.

We used the training set to develop our algorithm to differentiate between cases and controls. The best cutoff value for the dichotomous score was calculated based on the maximum Youden *J* statistic, i.e., *J* = max*_c_*(sensitivity*_c_* + specificity*_c_* - 1) for a cutoff *c* from the training set. This cutoff and the resulting predictor were then applied to the holdout set (Table 1).

#### Pathway and gene-enrichment analysis

To explore the biological significance of the CpGs included in our algorithm, we conducted a pathway and gene-enrichment analysis using the ***R*** package {missMethyl}, version 1.28.00 [(7)](https://sciwheel.com/work/citation?ids=148089&pre=&suf=&sa=0). Our aim was to explore associations between CpGs and biological pathways using gene ontology (GO) terms from the KEGG and GO databases that can be queried through {missMethyl}.

To perform the gene-enrichment analysis, we used the gometh function from {missMethyl} and applied the Benjamini-Hochberg method [(8)](https://sciwheel.com/work/citation?ids=6279401&pre=&suf=&sa=0) to account for multiple testing. The false discovery rate (FDR) was set to 0.05. To identify the most enriched pathways according to KEGG and GO terms, we used the topGSA function from {missMethyl}

All computational processing, modeling, and analysis were performed using the statistical programming language ***R***, version 4.1.3 [(9)](https://sciwheel.com/work/citation?ids=13672189&pre=&suf=&sa=0).

### Supplementary Text 2: Additional algorithm comparisons

#### Additional algorithm comparisons and sensitivity analyses

We compared the performance of our algorithm, to three alternative classification algorithms, by measuring the sensitivity, specificity and balanced accuracy in the holdout set and seronegative RA vs other arthritides subset (Supplementary Table 6). The alternative classification algorithms trained on age, sex and serology status (alternative #1), or age, sex, serology status and DNAm features (alternative #2), or DNAm features only (alternative #3). Our classification algorithm has the highest balanced accuracy in the full holdout set and when comparing seronegative RA to other arthritides. The alternative algorithm not using DNAm features has the lowest balanced accuracy in the seronegative RA vs other arthritides subset.

## [Supplementary References](https://sciwheel.com/work/bibliography)

[1. Aryee MJ, Jaffe AE, Corrada-Bravo H, Ladd-Acosta C, Feinberg AP, Hansen KD, et al. Minfi: a flexible and comprehensive Bioconductor package for the analysis of Infinium DNA methylation microarrays. Bioinformatics. 2014 May 15;30(10):1363–9.](https://sciwheel.com/work/bibliography/488308)

[2. Hofner B, Boccuto L, Göker M. Controlling false discoveries in high-dimensional situations: boosting with stability selection. BMC Bioinformatics. 2015 May 6;16:144.](https://sciwheel.com/work/bibliography/3491929)

[3. Friedman J, Hastie T, Tibshirani R. Regularization Paths for Generalized Linear Models via Coordinate Descent. J Stat Softw. 2010 Feb 21;33(1):1–22.](https://sciwheel.com/work/bibliography/171561)

[4. Shah RD, Samworth RJ. Variable selection with error control: another look at stability selection. J Royal Statistical Soc B. 2013 Jan;75(1):55–80.](https://sciwheel.com/work/bibliography/1509931)

[5. Barfield RT, Kilaru V, Smith AK, Conneely KN. CpGassoc: an R function for analysis of DNA methylation microarray data. Bioinformatics. 2012 May 1;28(9):1280–1.](https://sciwheel.com/work/bibliography/2040261)

[6. Zeng Y, Breheny P. The biglasso Package: A Memory- and Computation-Efficient Solver for Lasso Model Fitting with Big Data in R. R J. 2020;12(2):6.](https://sciwheel.com/work/bibliography/14859174)

[7. Ritchie ME, Phipson B, Wu D, Hu Y, Law CW, Shi W, et al. limma powers differential expression analyses for RNA-sequencing and microarray studies. Nucleic Acids Res. 2015 Apr 20;43(7):e47.](https://sciwheel.com/work/bibliography/148089)

[8. Benjamini Y, Hochberg Y. Controlling the false discovery rate: a practical and powerful approach to multiple testing. Journal of the Royal Statistical Society: Series B (Methodological). 1995 Jan;57(1):289–300.](https://sciwheel.com/work/bibliography/6279401)

[9. R Core Team. R: A language and environment for statistical computing. R Foundation for Statistical Computing. Vienna, Austria: R Foundation for Statistical Computing; 2022.](https://sciwheel.com/work/bibliography/13672189)
